# Treatment and outcome of CPD-associated peritonitis

**DOI:** 10.1186/1476-0711-5-6

**Published:** 2006-04-06

**Authors:** Laura Troidle, Fred Finkelstein

**Affiliations:** 1 New Haven CAPD, Renal Research Institute, New Haven, Connecticut, USA

## Abstract

CPD-associated peritonitis is a leading cause of morbidity and mortality for ESRD patients maintained on CPD therapy. The percentage of ESRD patients maintained on CPD therapy is declining. The reasons are unclear, but may be due to concerns about CPD-associated peritonitis.

The incidence of CPD-associated peritonitis has decreased largely attributed to technical advances and the identification of risk factors including exit-site infection, colonization with *Staphylococcus aureus *and depression.

The typical spectrum of organisms causing peritonitis include gram-positive organisms (67%), gram-negative organisms (28%), fungi (2.5%) or anaerobic organisms (2.5%). Culture-negative episodes do occur: up to 20% of the episodes of peritonitis in some series are culture-negative. The treatment of CPD associated peritonitis is rather standardized with current recommendations by the International Society of Peritoneal Dialysis universally adopted. Approximately 80% of the patients developing peritonitis will respond to antimicrobial therapy and remain on CPD therapy, while 10 to 15% of the patients require catheter removal and transfer to hemodialysis. Approximately 6% of the patients expire as a result of peritonitis. The outcome is different based on organism with gram-negative and fungal episodes having a worse outcome than gram-positive episodes.

The development of CPD-associated peritonitis can be linked to traditional risk factors such as exit-site infection and poor technique. Bacterial biofilm has also been suggested as a cause of peritonitis. Our current antimicrobial protocols may not permit adequate dosing to penetrate the biofilm and be a reason for recurrent or repeat episodes of peritonitis.

It is important that we improve our understanding of factors responsible for the development and outcome of CPD-associated peritonitis in order for this renal replacement therapy to remain a viable option for patients with ESRD.

## Introduction

The United States Renal Data System (USRDS) tracks all patients with end stage renal disease (ESRD) in the United States. The 2005 USRDS report indicated that there were 323, 926 prevalent patients in the United States maintained on dialysis therapy [1]. Only 25,825 (8.6%) of these prevalent patients were maintained on chronic peritoneal dialysis (CPD) therapy. This percentage of patients has been declining steadily over the last few years, from a peak of 15% in 1994 [2]. The reasons for the decline in the utilization of CPD therapy in the United States are not clear and have attracted considerable interest. Attention has focused on concerns with the structural organization of dialysis facilities, problems with the education of the patient with chronic kidney disease, problems with the training of nephrologists, and problems with technique failure, infection and patient mortality [3].

The high mortality rate of dialysis patients has been an area of much concern amongst nephrologists. Mortality attributed to infections, as well as hospital days related to infections have been noted to be significantly higher for patients maintained on peritoneal dialysis compared to patients maintained on hemodialysis [1]. CPD-associated peritonitis is the most common infection in CPD patients and has been noted to be not only a major cause of morbidity and mortality but also the leading cause of technique failure of patients maintained on CPD [4].

CPD-associated infections have thus received much attention and have been the subject of extensive investigation. Interest has been focused on strategies to reduce the incidence of infections, better understand the risk factors that predispose patients to infections, and develop better therapeutic strategies for treating these infections.

## Incidence and risk factors

The rate of CPD-associated peritonitis has decreased since CPD therapy was first described. Initial infection rates were more frequent than one infection per 12 patient months of therapy. Centers now routinely report infection rates of less than one episode in 24 patient months and as low as 1 episode in 60 patient months [5].

The improved rates have been attributed to technical advances as well as to the identification of risk factors associated with the development of CPD-associated peritonitis. For example, the development of the Ultra Twin Bag (Baxter) with its closed connectology immediately led to a reduction in the peritonitis rate when compared to the Ultra-Y-Set system that involved dual manual spiking procedures (1 episode/33.9 patient months vs. 1 episode/11.7 patient months) [6].

The association between a peritoneal catheter exit-site infection and the development of peritonitis has been well established. Twardowski and Prowant have developed explicit definitions of exit-site infections [7]. These clinicians suggest that aseptic techniques with nonirritating solutions be used and that catheters be immobilized. Furthermore, they have provided detailed protocols for caring for infected exit-sites. Others have advocated use of antibiotic creams, including mupirocin and gentamicin directly to the exit-site in the prevention of exit-site infection and peritonitis [8-10].

Nasal colonization with organisms such as *Staphylcoccus aureus *has also been linked to the development of peritonitis [11,12]. The Mupirocin Study Group randomized patients with nasal S aureus carriage to intranasal mupirocin or placebo with a significant reduction in exit-site infections with S aureus in the mupirocin treated group, yet no difference in the peritonitis rates [12]. The persistent nature of *Staphylococcus aureus *nasal colonization and the undesirability of applying mupirocin routinely to the nostrils lead to the use of mupirocin directly applied at the exit-site [8,9]. Bernardini et al prospectively randomized mupirocin applied to the exit-site versus oral rifampin with an equal benefit noted with either approach [8]. This group also showed that gentamicin cream was equally effective in preventing S aureus infections, but better at the prevention of gram-negative infections [10]. As a result, most centers have adopted either mupirocin or gentamycin cream as part of routine preventative care.

Depression, the most common psychological disorder among patients with ESRD has been linked to peritonitis in CPD patients. Our group has shown in a retrospective study that patients with more than one episode of peritonitis had significantly higher depression and anxiety than did patients with zero or one episodes of peritonitis [13]. We also showed in a prospective study that patients who manifested depressive symptoms, as indicated by a Beck Depression Inventory score of ≥ 11, had a significantly higher peritonitis rate than those patients with lesser amounts of depressive symptoms, after controlling for age, ethnicity and various co-morbid diseases [14]. Further studies are needed to clarify the impact of depression, that is potentially treatable, on the development of peritonitis.

## Definitions

*CPD-associated peritonitis *is defined as the presence of cloudy peritoneal effluent with 100 white blood cells/mm3 with greater than 50% polymorphonuclear cells. Abdominal pain and fever may or may not accompany these clinical and laboratory findings.

Episodes of peritonitis are determined to be *recurrent *or *relapsing *if the same organism with the same sensitivity pattern is isolated within the four-week period following the completion of a standard 2 week course of antimicrobial therapy. *Repeat *episodes of peritonitis are determined to occur if the patient develops peritonitis with the same organism and same sensitivity pattern greater than four weeks after the completion of antimicrobial therapy. Finkelstein et al using Spearman correlation testing found that 80% of CPD patients developing more than one episode of peritonitis had at least one repeat infection with the same organism [15]. In particular, there was a markedly increased statistically likelihood that both S. epidermidis and S. aureus would follow themselves as causative organisms for peritonitis.

*Polymicrobial *peritonitis is defined as the development of peritonitis with more than one organism and accounts for six percent of all episodes of peritonitis. Our group reviewed 80 episodes of polymicrobial peritonitis and found that 80% of the patients remain on CPD therapy which is typical for the more common single organism peritonitis [16]. And, surprisingly, underlying gastrointestinal disease was not common among the patients developing polymicrobial peritonitis. *Nosocomial *peritonitis, accounting for less than 1% of the episodes of peritonitis, is defined as the development of peritonitis in a hospital setting when no other infection was present at the time of admission (which may have lead to peritonitis) and there was no evidence that peritonitis was present at the time of admission. In our series, Nosocomial peritontitis developed in 5% of admissions of CPD patients over a one-year period with 42% of these patients expiring while being treated for the episode of peritonitis [17].

*Death due to peritonitis *has been defined as death due to sepsis, death occurring with a positive peritoneal dialysis culture, death within 14 days after onset of peritonitis or death occurring during hospitalization for any patient admitted with peritonitis [18]. Death attributed to peritonitis occurs in one to six percent of patients. It is worth noting that incidence of death associated with peritonitis has not improved despite a decrease in the peritonitis rate.

## Host defense in CPD-associated peritonitis

The peritoneal host defenses, including the peritoneal macrophages, mesothelial cells, fibroblasts and recruited neutrophils, are important in maintaining the peritoneum's response to infection. The peritoneal macrophage is the first responder along with the mesothelial cell; each stimulates a variety of cytokines and chemokines to attract neutrophils to the peritoneum. Several critical factors are then involved in the recruitment process to mediate an effective deployment of neutrophils [19].

It is well documented that the peritoneal dialysis solution itself can alter the immune response of the peritoneum [20]. Conventional dialysis solutions contain high lactate and glucose concentrations, a low pH, and glucose degradation products (that accumulate during the heat-sterilization process). These alter the peritoneal membrane's ability to initiate and sustain an immune response. Newer biocompatible solutions may offer a improved ability of the patient to combat bacterial infections. One study has suggested that patients using the newer, more biocompatible solutions have a significantly lower rate of peritonitis when compared to patients using traditional dextrose based solutions [21]

## Spectrum of organisms

The spectrum of organisms isolated among patients with CPD peritonitis is well described [22]. The typical spectrum of isolates include gram-positive organisms (67%), gram-negative organisms (28%), fungal (2.5%) or anaerobic organisms (2.5%). *Staphylococcus aureus *accounts for about 15% of the isolates and *Staphylococcus epidermidis *accounts for about 22% of the isolates. Recently, there has been a decline in the incidence of peritonitis caused by both *Staphylococcus aureus *and *Staphylococcus epidermidis *organisms, related in part to improved exit-site care and in part to newer techniques [5,22].

However, positive cultures are not obtained in all peritonitis episodes. In fact, in some series, up to 20% of the episodes of peritonitis are culture-negative [23]. The importance of adequate culturing techniques, therefore, cannot be overemphasized [24].

Antibiotic resistant organisms deserve mention in regard to their impact on outcomes of CPD-associated peritonitis. There have been reports of vancomycin resistant enterococci, vancomycin intermediate sensitive staphylococci and multi-drug resistant gram-negative organisms [25-27]. The poorer outcomes, in terms of morbidity and mortality, associated with these organisms deserves mention

## Treatment

Several antibiotic protocols exist for the treatment of CPD-associated peritonitis. The International Society of Peritoneal Dialysis has developed extensive guidelines for the treatment of CPD associated peritonitis [24]. [A complete listing of all antibiotic protocols can be obtained by contacting ] The cornerstones of the management of CPD associated peritonitis include broad empiric antibiotic coverage for both gram-positive and gram-negative organisms. Once the organisms and sensitivities are identified, appropriate adjustments in antibiotics should be made. Antibiotics are generally given intraperitoneally and continued for a minimum of two weeks. Persistent high white blood cell counts in the peritoneal fluid after 4 days of appropriate antibiotic therapy typically warrants consideration for catheter removal with temporary hemodialysis and intravenous antibiotics [24].

## Outcome

Several large outcome studies have noted that CPD associated peritonitis can be successfully cured with eradication of the infection and continuation of CPD therapy in 80–85% of the episodes of peritonitis. Approximately 10–15% of patients require catheter removal and transfer to hemodialysis during an episode of peritonitis [28,29].

In one to six percent of patients (quote studies), death has been associated with the peritonitis episode [28] Peritonitis, thus, may play a contributory role in the high mortality rate associated with dialysis therapy. For example, Fried et al noted a significant association of peritonitis with death in 15.8% of the CPD patients between 1979 and 1994 [18]. Inflammatory cytokines, including C-Reactive Protein, have been observed to be significantly elevated in the initial 48 hours of peritonitis and may remain elevated for weeks after the peritonitis episode [30]. This marker, linked to cardiovascular mortality, may provide a link between peritonitis and the associated mortality. Although peritonitis rates have decreased significantly in recent years, the outcome of peritonitis has not significantly improved despite the technical advances in CPD technology.

The outcomes observed for CPD-associated peritonitis varies depending on the organisms responsible for the infection. Goldie et al examined the outcome of 55 episodes of fungal peritonitis occurring in our unit [31]. 27 (49%) continued CPD therapy following the completion of anti-fungal therapy and the removal of the peritoneal catheter, 17 (31%) transferred to hemodialysis and 11 (20%) expired. Both our group and Bunke et al noted that patients developing gram-negative peritonitis had a worse outcome than did patients developing gram-positive peritonitis [28,29]. In our analysis 97% of the patients developing gram-positive peritonitis remained on CPD therapy at 2 weeks while only 73% of patients developing gram-negative peritonitis remained on CPD therapy (p < 0.05). Patients developing gram-negative peritonitis were significantly more likely than patients developing gram-positive peritonitis to be hospitalized (74% vs 24%, p < 0.001) and expire within six months after the onset of peritonitis (21% vs 9%, p < 0.05). Peritonitis with *Staphylococcus aureus *also contributes to the poor outcome associated with peritonitis. Krishnan et al noted that only 68.6% of Staphylococcus aureus peritonitis resolved with continued CPD therapy compared to 94.2% of the other gram-positive episodes resolved [32].

The indwelling silastic peritoneal catheter has been implicated as a source and nidus of infection. It is necessary to remove the peritoneal catheter for certain types of peritonitis, such as those caused by Pseudomonas, fungi, mycobacterium and vancomycin-resistant enterococci [24]. In addition, it has been recommended that the peritoneal catheter be removed for patients who develop recurrent peritonitis with the same organism, patients who fail to respond to appropriate antibiotic therapy with four days, and patients who develop repeat infections [24].

Technique failure for patients who undergo peritoneal catheter removal is high, since it is often difficult to re-establish a viable peritoneal access. For example, our group noted a guarded outcome following the removal of the peritoneal catheter for peritonitis [33]. Only 47% of the patients were able to successfully return to CPD therapy. Furthermore, 23% of the patients died following catheter removal and another 26% died in the first year following the reinsertion of the peritoneal catheter. Similarly, Szeto et al noted that only 51% of the patients who had the peritoneal catheter removed during an episode of peritonitis had the catheter reinserted to continue CPD therapy and that only 25% of these patients remained on CPD therapy 24 months after catheter removal [34].

## CPD modality and peritonitis

There are two types of CPD therapy: continuous ambulatory peritoneal dialysis (CAPD) and continuous cycling peritoneal dialysis (CCPD). The former modality involves manual dialysis exchanges performed throughout the day (usually three to five) while the latter modality is performed continuously for approximately 8 to 10 hours at night using an automated cycling device. CCPD therapy is performed on the vast majority of CPD patients in the United States.

There has been considerable debate regarding the effect of either modality on the development of peritonitis. The European Automated Peritoneal Dialysis Outcomes Study reported higher rates of peritonitis for patients maintained on CAPD compared to patients maintained on CCPD therapy [35]. Both our group and Yishak et al reported similar rates of peritonitis for patients maintained on either modality [36,37]. And, Oo et al, using a USRDS database between 1994 and 1997 noted that CAPD therapy was associated with a lower risk of development of a first episode of peritonitis after nine months of CPD therapy [38].

Whether there are differences in the development and outcome of peritonitis for patients maintained on CAPD and CCPD is debatable. Yishak et al did note a trend toward more adverse events among patients maintained on CCPD compared to patients maintained on CAPD (12% vs 6%, p = .21) [37]. Why might there exist a difference in outcomes?

While pharmacokinetic data concerning a multitude of antimicrobial agents used to treat peritonitis for patients maintained on CAPD has been well defined, there is a little scientific data to support the ISPD recommendations for treating patients with CCPD [24,38,39]. The ISPD recommends once-daily intraperitoneal (IP) administration of antimicrobial agents for patients maintained on CCPD or CAPD, administering the antibiotics during a long dwell exchange. But because of augmented antibiotic clearance with CCPD and the use of rapid exchanges during nighttime cycling, adequate antibiotics levels may not be achieved in the PD fluid during CCPD therapy (Manley et al Semin Dial 15:418, 2002). For example, Manley et al found once-daily loads of cefazolin and ceftazidime resulted in levels below target MIC during cycling exchanges [40]. Thus, some investigators have suggested that all patients developing peritonitis be converted to CAPD or that the rate of cycling be slowed while patients are being treated for peritonitis.

## Biofilm

Some episodes of peritonitis can be linked to poor patient technique or a concomitant exit-site infection. But, many episodes of peritonitis appear to be unrelated to obvious causes. Some episodes, particularly those episodes involving S. aureus and S. epidermidis, recur or repeat despite standard antibiotic therapy [15]. Furthermore, other episodes of peritonitis do not resolve unless the peritoneal catheter is removed. Is there a nidus of infection that persists? Is there a role for bacterial biofilm?

Costerton first described a sessile population of bacteria that coexisted with free-floating, planktonic bacteria in 1978 [41]. It is known that these planktonic bacteria attach themselves to a foreign body such, as the silastic peritoneal catheter [42]. These attached bacteria become a sessile population with an impenetrable glycocalyx matrix coat. Bacterial cells within the sessile population communicate via a complex quorum sensing network which is necessary for the production of biofilm [41,43]. Biofilm, thus, is an example of a viable bacterial ecosystem.

Antibiotic penetration into this complex ecosystem presents special problems. Do our current antimicrobial therapies allow for penetration into the biofilm? Sepandj examined this by studying the minimum biofilm erradication concentration (MBEC) of a first generation cephalosporin for coagulase-negative staphylococci [44]. This group noted that the MBEC was particularly high, and, that the percentage of resistant organisms increased when patients experienced repeat infections.

Yet, Costerton et al note that the poor penetration of the antibiotic into the complex biofilm matrix was not the only hurdle to overcome in the eradication of biofilm [41]. This group also has noted that bacteria within the biofilm can differentiate into "protected phenotypic states" permitting these sessile bacteria to further resist standard antibiotic strategies.

We modified Costerton's techniques to examine biofilm via confocal scanning light microscopy on the peritoneal catheter [45]. A total of 10 peritoneal catheters were removed and subsequently examined for biofilm. Biofilm was identified on all ten catheters. The peritoneal catheters were removed because of peritonitis in eight patients and because the peritoneal catheter was no longer needed in two patients.

Table 1 identifies the individual organisms identified within the biofilm of these removed catheters. All eight of these catheters had the same organism isolated form the biofilm that was isolated from the peritoneal effluent in a previous episode of peritonitis.

**Table 1:** 

**Organism**	**# Catheters Involved**	**Biofilm Organism Same?**
*Staphylococcus aureus*	1	Yes
*Enterococcus*	1	Yes
*Pseudomonas aeruginosa*	3	Yes
*Enterobacter*	1	Yes
*Bacteroides fragilis*	1	Yes
*Escherichia coli*	1	Yes
*Candida*	1	Yes

These data suggest that bacterial biofilm does develop on the catheter and does represent a concern for CPD patients in terms of our ability to eradicate the infection and in terms of the development of recurrent or repeat peritonitis episodes. It is necessary that we better understand the complex biological ecosystem of biofilm so that better strategies can be developed to both eradicate the biofilm. Studies need to be done to determine if our current antibiotic strategies result in antibiotic penetration within the biofilm matrix.

## Conclusion

The percent of ESRD patients maintained on CPD therapy has been declining. The reasons for this are unclear, but may in fact be related to concerns about CPD-associated peritonitis. Although the infection rate has declined in recent years as the technology of CPD has advanced, peritonitis remains a leading cause of both technique failure and mortality for patients maintained on CPD therapy. It is important that we improve our understanding of those factors that are responsible for peritonitis and that we develop better treatment paradigms for the management of these infections.

**Figure 1:**
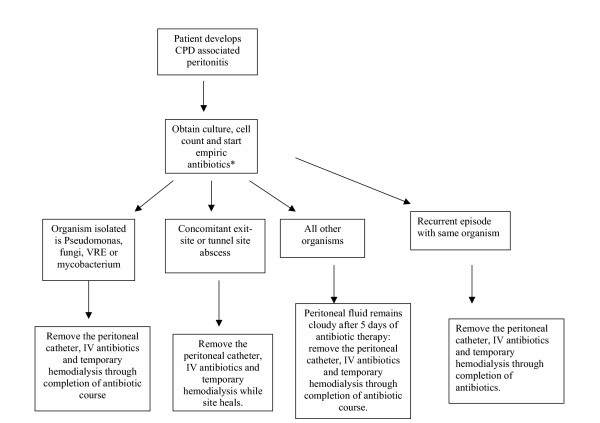
Management of the Peritoneal Catheter During Peritonitis.
